# Mechanism and clinical application of thymosin in the treatment of lung cancer

**DOI:** 10.3389/fimmu.2023.1237978

**Published:** 2023-08-28

**Authors:** Yafeng Liu, Jibin Lu

**Affiliations:** Department of Thoracic Surgery, Shengjing Hospital of China Medical University, Shenyang, China

**Keywords:** thymosin, lung cancer, therapy, thymosin alpha 1, thymalfasin, thymopentin, immunotherapy

## Abstract

Cancer is one of the leading causes of death worldwide. The burden of cancer on public health is becoming more widely acknowledged. Lung cancer has one of the highest incidence and mortality rates of all cancers. The prevalence of early screening, the emergence of targeted therapy, and the development of immunotherapy have all significantly improved the overall prognosis of lung cancer patients. The current state of affairs, however, is not encouraging, and there are issues like poor treatment outcomes for some patients and extremely poor prognoses for those with advanced lung cancer. Because of their potent immunomodulatory capabilities, thymosin drugs are frequently used in the treatment of tumors. The effectiveness of thymosin drugs in the treatment of lung cancer has been demonstrated in numerous studies, which amply demonstrates the potential and future of thymosin drugs for the treatment of lung cancer. The clinical research on thymosin peptide drugs in lung cancer and the basic research on the mechanism of thymosin drugs in anti-lung cancer are both systematically summarized and analyzed in this paper, along with future research directions.

## 1 Introduction

Cancer is one of the main causes of death on a global scale (1). Cancer has been acknowledged to be an increasing public health burden (2). Lung cancer (LC) is the leading cause of cancer deaths worldwide, and it has a relatively high incidence (3). With the popularity of low-dose computed tomography as an early screening test, more and more early lung cancers, particularly early lung adenocarcinoma (LUAD), are being detected (4, 5). LC includes non-small cell lung cancer (NSCLC) and small cell lung cancer (SCLC), and NSCLC contains numerous pathological types such as lung squamous cell carcinoma (LUSC) and lung adenocarcinoma, of which LUAD is the most prevalent pathological type of LC (6). After surgical resection, early-stage LC, especially LUAD including ground-glass opacity components, has a very favorable long-term survival outcome (7, 8). In recent years, the emergence and development of targeted therapy and immunotherapy as well as the application of these methods to neoadjuvant therapy, have also greatly improved the prognosis of LC patients (9–12). Even though the survival rate of lung cancer patients has gradually increased, the survival of patients with advanced lung cancer is not encouraging, and the 5-year survival rate of patients with advanced LC is even lower than 20% (13). Today, there are still issues such as drug resistance, negative gene mutations, and negative immunotherapy targets for LC patients or poor treatment effects and incapacity to tolerate the toxic side effects of treatment. Therefore, for LC, whether it is the discovery of innovative, safe, and effective treatment options or the investigation of auxiliary therapeutic drugs, the need is still very urgent.

**Figure 1:**
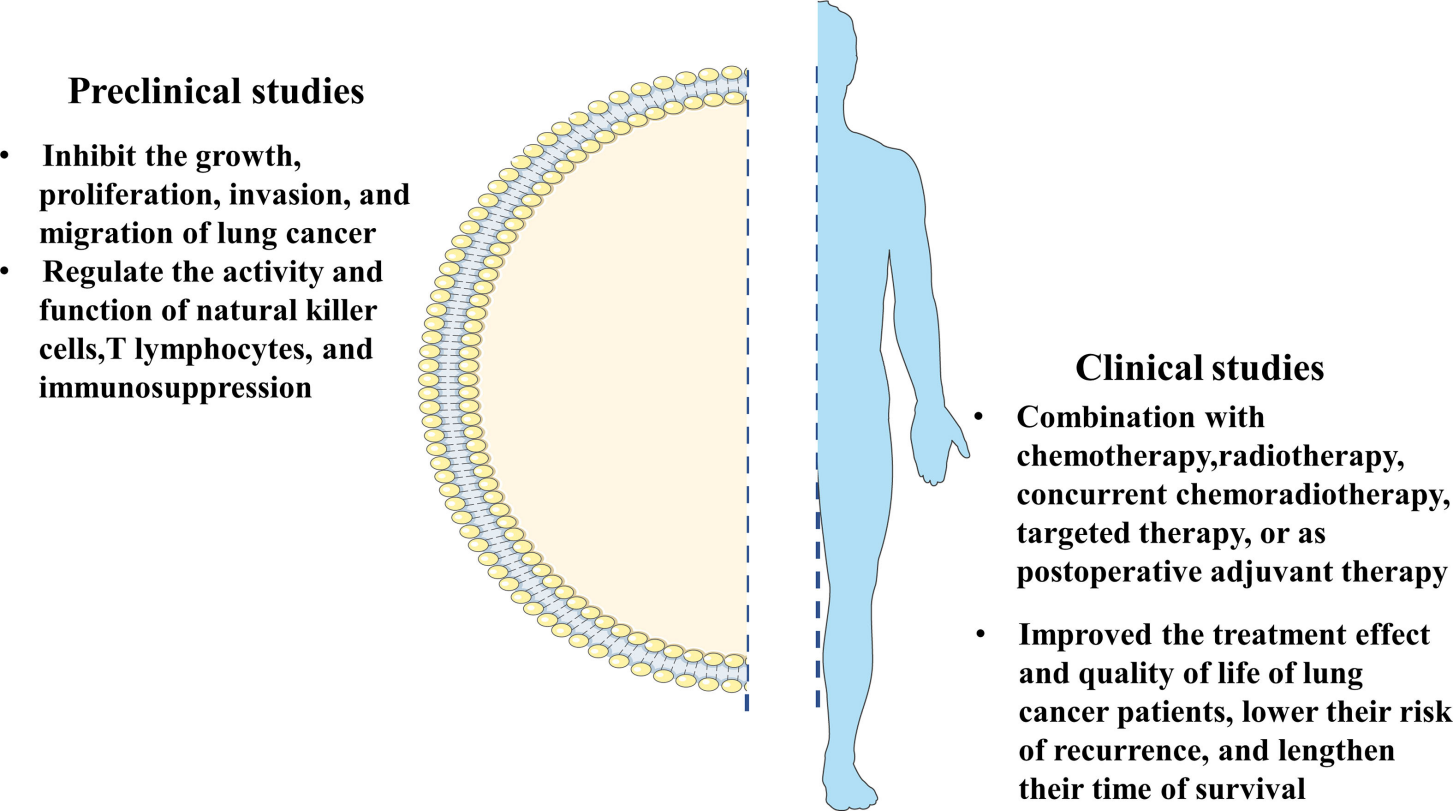
The summary of clinical research and the basic research on the mechanism of thymosin drugs in anti-lung cancer.

The thymus gland is an organ in the body that has immunological and endocrine functions (14, 15). In the 1960s, researchers extracted hormones such as thymosin from the thymus gland, based on this discovery, the primary active substance of thymosin, thymosin fraction 5, was then isolated and further separated and purified as thymosin II and thymosin alpha1 (Tα1) (16). Thymosin is a class of physiologically active polypeptides released by the thymus gland, and the most widely used thymosin drugs in clinical practice mainly include thymosin, thymosin alpha-1, and thymopentin (17). Thymosin is a mixture of peptides extracted from the thymus glands of animals. Tα1, also known as thymalfasin, is a medication completely consistent with human Tα1, which is a small molecule bioactive polypeptide obtained by isolation and purification of thymosin component 5, made up of 28 amino acids and a molecular weight of 3,018 Da (18, 19). The immunoreactive center of thymosin II, a polypeptide fragment composed of 5 amino acids, located in the 32-36th position of the polypeptide, is called thymopentin (20, 21). The most common dosage forms of thymosin drugs include thymosin injection, Tα1 injection, thymopentin injection, and thymosin enteric-coated tablets/capsules. Thymosin drugs have an immunomodulating and boosting immunity function, suited for treating individuals with a compromised immune status caused by sepsis, and other illnesses (22–24). Additionally, thymosin drugs are also frequently used in the treatment and adjuvant treatment of cancer (25–27). There have been numerous clinical studies and basic mechanism studies that have demonstrated the effectiveness of either thymosin monotherapy or combination therapy for the treatment of LC (26). This fully illustrates the potential of thymosin for the treatment of LC. However, there is currently a lack of systematic summarization of thymosin in the treatment of LC. Therefore, this study organizes and analyzes basic and clinical research in this field (**Figure 1**), to clarify the current situation and explore future research directions.

## 2 Summary of the mechanism of thymosin in the treatment of LC

**Figure 2:**
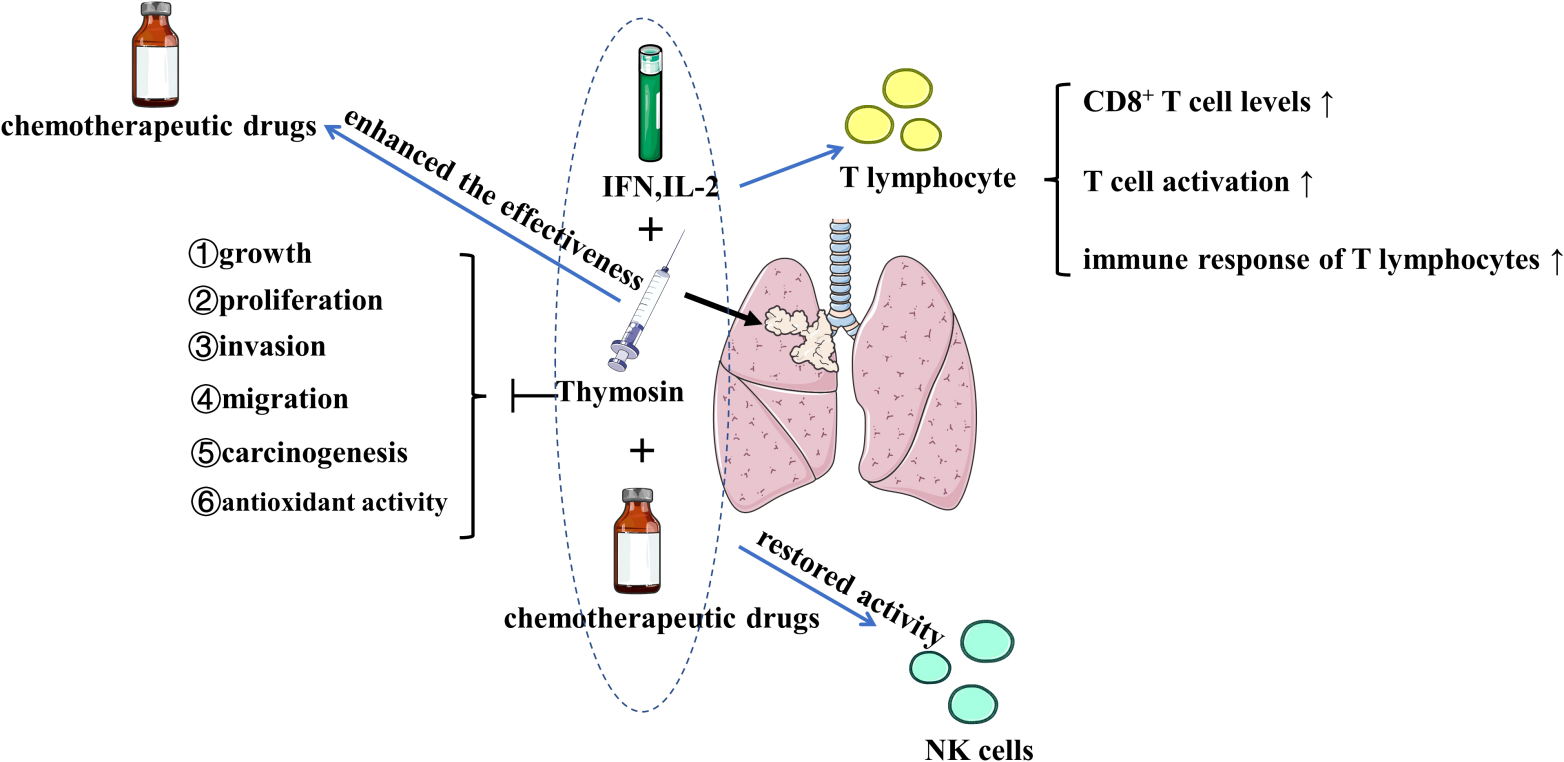
The effects of thymosin monotherapy and combination therapy (with chemotherapeutic drugs, IFN or IL-2) on the treatment of lung cancer Thymosin monotherapy and combination therapy inhibit the growth, proliferation, invasion, migration, carcinogenesis, and antioxidant activity of lung cancer. Thymosin plus IFN and IL-2 exert positive immunomodulatory effects on T lymphocyte by upregulating CD8^+^ T cell levels, increasing T cell activation, and enhancing the immune response of T lymphocytes. Thymosin restores the activity of NK cells, enhances the effectiveness of chemotherapeutic drugs. IFN, interferon; IL-2, interleukin-2; NK, natural killer. The figure is partly generated using Servier Medical Art provided by Servier, licensed under a Creative Commons Attribution 3.0 Unported License.

**Figure 3:**
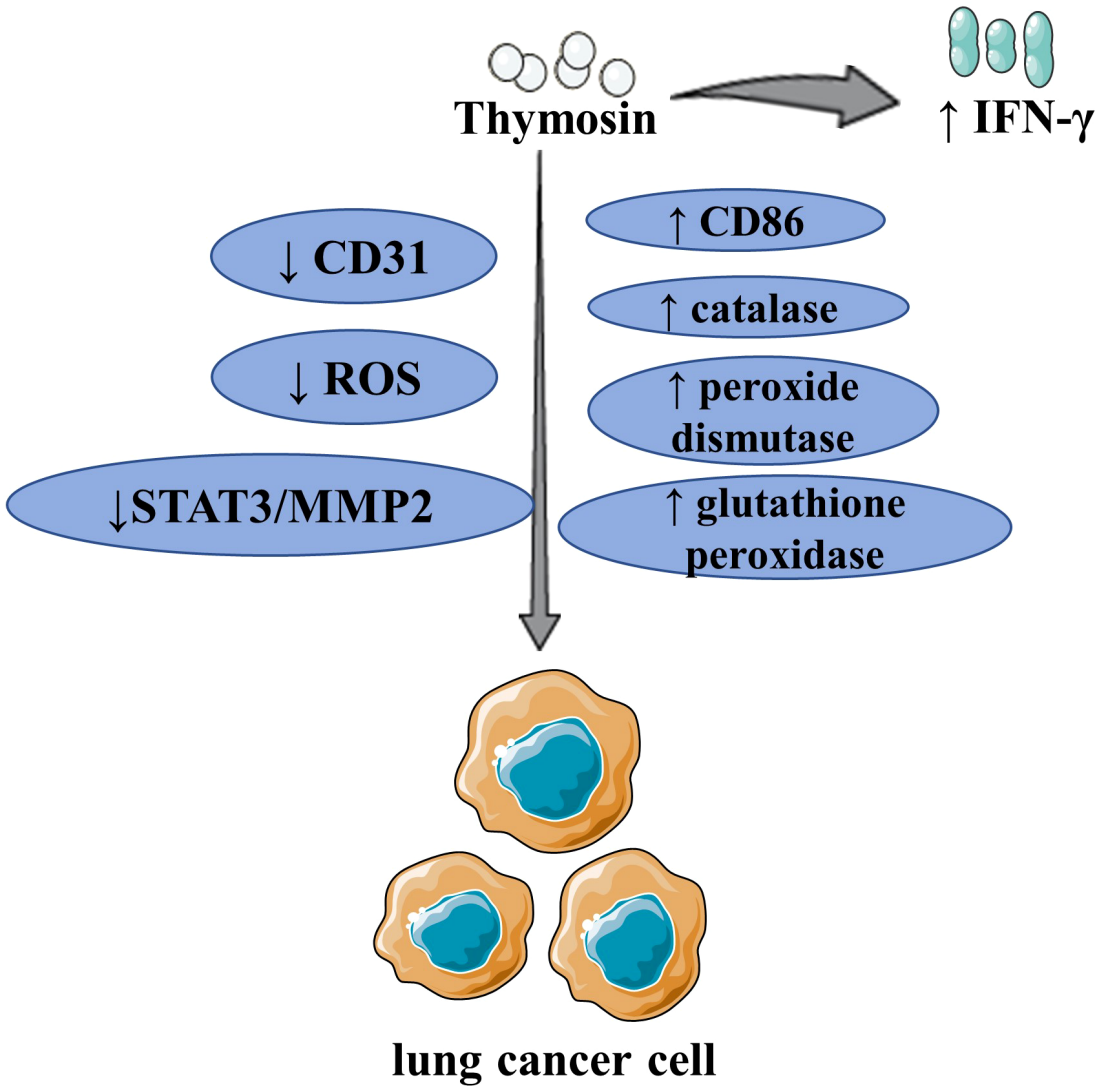
The major mechanism of thymosin in the treatment of lung cancer By upregulating IFN-γ and CD86, as well as inhibiting CD31, thymosin inhibited the growth of LCLC/LUAD and boosted the immune response of T lymphocytes. Thymosin inhibited the proliferation, migration, and antioxidant activity of LC by inhibiting the expression of ROS, upregulating catalase, peroxide dismutase, and glutathione peroxidase. Thymosin increased CD8^+^ T cell levels, inhibited the growth of LUAD. Thymosin inhibited the invasion and migration of LC by inhibiting STAT3 and then MMP2. ROS, reactive oxygen species; IFN-γ, interferon-γ; STAT3, signal transducer and activator of transcription 3; MMP2, matrix metalloproteinase 2; LCLC, large cell lung cancer; LUAD, lung adenocarcinoma; LC, lung cancer. The figure is partly generated using Servier Medical Art provided by Servier, licensed under a Creative Commons Attribution 3.0 Unported License.

Many scholars have advanced the mechanism of thymosin in LC and undertaken in-depth and extensive research. Numerous mechanisms have been identified so far, including inhibition of LC growth, proliferation, invasion, and migration, and immunomodulatory effects that regulate the activity and function of natural killer cells, T lymphocytes, and immunosuppression, to exert anti-LC effects. The mechanism and effects of thymosin in the treatment of LC are summarized in detail in **Figures 2**, **3**, and **Table 1**.

**Table 1:** The major mechanism and effects of thymosin in the treatment of lung cancer.

Drug/gene	Major mechanism and effects	References
Tα1	STAT3-MMP2 ↓, ROS↓, catalase↑, peroxide dismutase↑, glutathione peroxidase↑; Prevented lung carcinogenesis, and inhibited the growth, proliferation, invasion, migration, and the antioxidant activity of lung cancer	(28–32),
Tα1 and IFN/IL-2	Inhibited the growth of lung cancer, restored NK cell activity	(33)
Tα1 and IFN α/β and cyclophosphamide	Inhibited the growth of lung cancer, enhanced the effectiveness of cyclophosphamide and restored NK cell activity	(34)
Tα1 and IL-2 and cyclophosphamide	Inhibited the growth of lung cancer, enhanced the effectiveness of cyclophosphamide	(35)
Tα1 and αβ-IFN and cyclophosphamide	Restored NK cell activity	(36)
Tα1 and gemcitabine	ARG1↑, TLRs/MyD88↑; Increased CD8+ T cell levels, inhibited the growth of lung adenocarcinoma, promoted the activation of myeloid-derived suppressor cells	(37)
Tα1-iRGD	CD86↑; Inhibited the growth of large cell lung cancer, promoted T cell activation	(38)
Tα1-RGDR	IL-2↑, IFN-γ↑, CD31↓; Inhibited the growth of large cell lung cancer/lung adenocarcinoma, boosted the immune response of T lymphocytes	(39)
Tβ4 (silencing)	Notch1↓; Inhibited proliferation and invasion of lung adenocarcinoma	(40)

Tα1, thymosin alpha1; STAT3, signal transducer and activator of transcription 3; MMP2, matrix metalloproteinase 2; ROS, reactive oxygen species;IFN, interferon; IL-2, interleukin-2; ARG1, arginase 1; TLRs, toll-like receptors; MyD88, myeloid differentiation primary response gene 88; RGD, arginine-glycine-aspartic acid; Tβ4, thymosin β4.

### 2.1 Inhibit the growth, proliferation, invasion, and migration of LC

At present, a large number of studies have confirmed that thymosin drugs in combination with other drugs or monotherapy have the effect of inhibiting the growth of LC (28–30, 33–35). Both *in vitro* and *in vivo* experiments have demonstrated that Tα1 monotherapy can significantly inhibit the growth of NSCLC (28). Chemoprevention, a method, and strategy to reduce cancer incidence and mortality, is defined as the use of natural or synthetic substances, biological agents, and other pharmacological agents in the early stages of cancer to achieve the purpose of impeding, arresting, or even reversing the development of cancer (41). Chemoprevention has a wide range of applications in the field of LC and is one of the potential therapeutic options to reduce the risk of LC (42). According to studies, Tα1 can be used as a chemopreventive drug that can effectively prevent the occurrence of LUAD, and Tα1 also has a direct inhibitory effect on the growth of LUAD (29, 30). In addition to the monotherapy of thymosin drugs, many scholars have also paid attention to the effect and mechanism of thymosin drugs combined with other drugs on LC, these studies shown that Tα1 in combination with interferon (IFN) α/β or interleukin-2 (IL-2) can inhibit LUAD growth and that Tα1 has a synergistic effect with IFNα/β or IL-2, and the combination has a better effect of inhibiting tumor growth than Tα1 monotherapy (33–35). The dysregulation of the JAK/STAT pathway is closely related to LC and other cancers, STAT3 plays an important role in the JAK-STAT signaling pathway, and STAT3 is primarily involved in tumor growth, differentiation, migration, and invasion regulation (43, 44). A kind of matrix metalloproteinase named MMP2 can degrade type IV and type V collagen and elastic fibers, thereby destroying the basement membrane and extracellular matrix, and promoting tumor invasion and metastasis (45). Bo et al. discovered that Tα1 inhibited the transcription of MMP2 by inhibiting phosphorylation of STAT3, resulting in a decrease in MMP2 protein expression and inhibiting the migration and invasion of NSCLC cells with programmed cell death ligand 1 (PD-L1) high-expressing, which suggests that Tα1 may inhibit the STAT3-MMP2 signaling pathway by stimulating PD-L1 (31). One of the main components produced by oxidative stress in the body is reactive oxygen species (ROS), which has the role of driving the development of cancer and other diseases, and the increase of ROS can promote the growth, migration, and invasion of tumors (46). ROS is also one of the main causes of LC occurrence and progression (47). A study has confirmed that Tα1 has the effect of inhibiting the proliferation and migration of LUAD cells, and Tα1 also can inhibit the antioxidant activity of LUAD cells by reducing ROS levels and increasing the activity of antioxidant enzymes like catalase, superoxide dismutase, and glutathione peroxidase (32). Thymosin β4 (Tβ4) is also thought to be associated with LC in addition to Tα1. Tβ4, a polypeptide with 43 amino acids that is distributed in various tissues of the human body and is one of the main actin regulators in the human body, makes up the largest proportion of β-thymosin (48, 49). According to Huang et al., Tβ4 was overexpressed in NSCLC, and after Tβ4 was silenced, the activation of Notch1 was inhibited, which inhibited the proliferation and migration, and invasion of LUAD cells (40).

### 2.2 Immunomodulatory effects

#### 2.2.1 Natural killer cell

Natural killer (NK) cells are important immune cells in the human body and the main effector cells of innate immunity. The use of anti-tumor immunotherapy based on NK cells is being investigated (50). Some of the side effects of chemotherapy are related to its suppression of the immune system. In animal models of LC after chemotherapy, NK cell activity was restored simultaneously with the inhibition of LC growth and an improvement in the prognosis for LC which may be related to the role of NK cells in inhibiting LC growth and metastasis (33, 34, 36). The benefits of Tα1 and IFNs in animal models of LC after chemotherapy also disappeared after the consumption of NK cells, further proving that the effectiveness of Tα1 in LC treatment is likely to be obtained by modulating NK cells (35).

#### 2.2.2 T lymphocytes

T lymphocytes are the main components of lymphocytes and play a crucial role in cellular immunity (51). For instance, the success of anti-T lymphocyte inhibitory receptor therapy for PD-L1 and cytotoxic T-lymphocyte associated antigen-4 (CTLA-4) in LC also highlights the important role of CD8^+^T cells in anti-tumor immunotherapy (52, 53). In animal models with LUAD, Tα1 monotherapy, and Tα1 in combination with gemcitabine have both been shown to increase CD8^+^T cell levels (37). The anti-tumor positive effects of Tα1 and IFNs were counteracted when T lymphocytes are consumed, and after the application of Tα1 and IFNs, there was a significant T lymphocyte infiltration in the necrotic area of LC (35). These observations all confirm the idea that the regulation of T lymphocytes is one of how Tα1 exerts anti-LC effects. Targeted drug ligands like the arginine-glycine-aspartic acid (RGD) peptide are a class of polypeptides that have garnered a lot of attention for their use in the transportation of anti-cancer drugs (54). RGD peptide can target drug delivery into tumors by binding to integrins, such as αvβ3 and αvβ5, which are highly expressed in tumor blood vessels (55). RGD peptides with high affinity with αv integrins and neuropilin-1 are known as iRGD peptides (56). It has been demonstrated in the large cell lung cancer (LCLC) model that the use of iRGD and Tα1 fusion to form modified Tα1, or Tα1-iRGD, can promote T cell activation, can restore thymus index and spleen index, and also activate T lymphocytes by increasing the expression of CD86 to exert their immune effect (38). In addition, compared to Tα1, the modified Tα1 has better immunomodulatory activity and stronger LC inhibition and also exhibits lower tumor vascular density, which is associated with the improved targeting of the modified Tα1-iRGD. A study also confirmed that binding Tα1 to RGD peptides compensated for the lack of tumor targeting of Tα1 (39). RGDR can bind to highly expressed αvβ3 and NRP-1 domains on the tumor surface because it includes the RGD sequence. Integrin αvβ3 is an integrin protein closely related to tumor angiogenesis and specifically able to recognize RGD sequences (55). NRP-1 is a transmembrane glycoprotein that is widely expressed in tumor tissues and plays a key role in tumor angiogenesis (57, 58). Therefore, Tα1-RGDR formed by fusing Tα1 with RGDR can achieve a good tumor-targeting effect. Tα1-RGDR showed stronger anti-LUAD and anti-LCLC effects than the Tα1, and it can boost the immune response of T lymphocytes by increasing IL-2 and IFN-γ levels and promoting the invasion of CD4^+^T cells and CD8^+^T cells into tumor tissues to exert anti-LC effects.

#### 2.2.3 Immunosuppression

Tα1 has potent anti-LC and immunomodulatory effects when used alone or in combination with other medications. However, Tα1 and chemotherapy drugs show a stronger anti-lung cancer and immunomodulatory effect than Tα1 alone, which may be related to the synergistic effect of Tα1 with other medications. A study showed that neither Tα1 nor gemcitabine (GEM) alone could inhibit the growth of LUAD, but when combined Tα1 with GEM showed higher levels of CD8^+^T cells and the ability to significantly inhibit LUAD growth, indicating that GEM and Tα1 do have a certain synergistic effect (37). The study also discovered that Tα1 monotherapy can upregulate Arginase 1 (ARG1) to activate myeloid-derived suppressor cells (MDSCs) in animal models of LUAD to produce inhibitory anti-tumor immune effects, which may account for the limited effect of Tα1 monotherapy. According to some reports, Tα1 interacts with toll-like receptors (TLRs) and activates MyD88 signaling in dendritic cells, and upregulation of ARG1 relies on the TLRs/MyD88 signaling pathway (59, 60). That is, Tα1 increases the expression of ARG1 by activating TLRs/MyD88 signaling pathways, thereby activating the immunosuppressive ability of MDSCs.

## 3 Summary of the clinical studies of thymosin in the treatment of LC

Tα1 is frequently used in clinical practice because it acts as an immunomodulator, boosting the body’s defenses and enhancing the quality of life for LC patients (61). Numerous clinical studies have demonstrated that the use of Tα1 in combination with chemotherapy, radiotherapy, concurrent chemoradiotherapy (CCRT), targeted therapy, or as postoperative adjuvant therapy can significantly increase the clinical efficacy of LC patients, lower their risk of recurrence, and lengthen their time of survival. All the clinical studies mentioned in the paper are summarized in **Table 2**.

**Table 2:** The clinical effects of thymosin in the treatment of lung cancer.

The usage of thymosin	Drug	Conclusion	References
	Tα1 and low-dose IFN-α	Increased the response rates of chemotherapy, prolonged the time to progression	(62)
Combination with chemotherapy	Tα1	Increased ORR, TCR, 1-year survival rate and QOL	(63)
	Tα1/thymopentin	Increased ORR, QOL, one-year OS rate, DCR, CD3^+^ T cell levels, CD3^+^CD4^+^ T cell levels, NK cell numbers, and the proportion of CD4^+^/CD8^+^ T cells, decreased the risk of neutropenia, thrombocytopenia, and gastrointestinal reactions	(64)
Combination with radiotherapy	Tα1	Improved OS, DFS, and restored T helper cell levels	(65)
Combination with concurrent chemoradiotherapy	Tα1	Decreased the incidence of grade 2 or higher RP and grade 3 to grade 4 lymphopenia, increased total lymphocyte count, and decreased CRP	(66)
Combination with targeted therapy	Tα1/thymopentin	Improved median OS and median PFS, restored the number of CD3^+^ T cells and CD3^+^CD4^+^T/CD3^+^CD8^+^ T subsets	(67)
Postoperative adjuvant therapy	Tα1	Increased 5-year OS rate and 5-year DFS rate	(68)

Tα1, thymosin alpha1; IFN, interferon; ORR, overall response rate; TCR, tumor control rate; QOL, quality of life; DCR, disease control rate; NK, nature killer; OS, overall survival; DFS, disease-free survival; RP, radiation pneumonitis; CRP, C-reactive protein; PFS, progression free survival.

### 3.1 Combination with chemotherapy

Chemotherapy is a common treatment for patients with advanced LC and those with early-stage, high-risk LC (69, 70). Compared to the control group of NSCLC patients receiving only ifosfamide chemotherapy, patients receiving Tα1 and low-dose interferon-α in addition to chemotherapy had higher response rates (33% vs. 10%) and longer times to progression (TTP) (p=0.0059), according to a phase II clinical study. In the chemotherapy group, CD4^+^, CD8^+^, and NK cell counts significantly declined. Combination therapy with Tα1 reduced the toxicity of chemotherapy while leaving lymphocyte counts essentially unchanged from before chemotherapy (62). The effectiveness of thymosin in combination with chemotherapy was assessed in a meta-analysis of 10 randomized controlled trials (RCTs) in a total of 724 patients with advanced (stage III, IV) NSCLC regimens, primarily cisplatin plus vinorelbine (NP) regimens and gemcitabine plus cisplatin (GP) regimens. Thymosin plus NP was found to increase overall response rate (ORR) (odds ratio (OR) 1.86) compared with the control group using NP-only chemotherapy, and the addition of thymosin also increased tumor control rate (TCR) (OR 3.06), 1-year survival rate (OR 3.05) and improved the quality of life (QOL) (OR 3.39). Thymosin and GP-containing chemotherapy showed superior ORR (OR 1.67), TCR (OR 2.38), and QOL (OR 3.84) compared to GP-only chemotherapy. These findings imply that for patients with advanced NSCLC, thymosin plus NP/GP chemotherapy is preferable to NP/GP chemotherapy alone (63). Another meta-analysis included 27 RCTs involving a total of 1925 patients with stage IIIa-IV NSCLC, of whom 976 received combination therapy with synthetic thymic peptides (sTPs) (including Tα1 and thymopentin) and chemotherapy and 949 received chemotherapy alone to evaluate the clinical effects of sTPs plus chemotherapy (64). In comparison to chemotherapy, the use of sTPs significantly improved QOL [relative risk (RR) = 2.05, P<0.00001], ORR [RR = 1.28, P = 0.0001], and one-year overall survival (OS) rate [RR = 1.43, P = 0.001], as well as increased disease control rate (DCR) [RR = 1.10, P = 0.02]. The combination of sTPs and chemotherapy demonstrated that the combination therapy was clinically effective. Combination therapy with sTPs and chemotherapy increased CD3^+^T cell levels [P<0.00001], CD3^+^CD4^+^T cell levels [P<0.00001], NK cell numbers [P<0.00001], and the proportion of CD4^+^/CD8^+^T cells [P<0.00001] at the lymphocyte level, and it can lessen the immunotoxic effects of chemotherapy when combined with sTPs. Additionally, the risk of chemotherapy-related side effects like neutropenia (RR=0.75, P=0.04), thrombocytopenia (RR=0.68, P=0.0002), and gastrointestinal reactions (RR=0.62, P<0.00001) was decreased by sTPs. To compare the two sTPs of Tα1 and thymopentin, further subgroup analysis was done. The results showed that Tα1 could significantly increase ORR [RR: 1.32, P=0.0003] and DCR [RR: 1.11, P=0.02], but that there were no statistically significant differences in OR and DCR in the thymopentin subgroup. Tα1 and thymopentin could also increase the level of peripheral blood lymphocytes. It was demonstrated that Tα1 could boost clinical effectiveness while also boosting immune function.

### 3.2 Combination with radiotherapy

Although radiation therapy is one of the most effective treatments for advanced lung cancer, it invariably results in side effects, such as immune response (71). An RCT study analyzed the effect of synthetic Tα1 on immune recovery properties in NSCLC patients after radiotherapy (65). The findings revealed that thymosin treatment following radiotherapy significantly improved OS (P=0.009) and disease-free survival (DFS) (P=0.04) compared with placebo, and thymosin restored the decrease in T helper cell levels brought on by radiotherapy (p=0.04). This proved that Tα1 can both enhance the efficacy of radiotherapy and reverse immunosuppression brought on by radiotherapy. The experimental group received two regimens of subcutaneous injection twice a week with Tα1 900 mg/m2 (body surface area) or continuous thymosin 900 mg/m2 subcutaneous injection once daily for 14 days, followed by twice-weekly subcutaneous injection. The study evaluated the difference in efficacy between different dosing regimens, with the control group receiving a placebo subcutaneous injection regimen. The results showed that the control group showed shoddy results. In the experimental group, there was no discernible difference between the two regimens, indicating that both regimens might be practicable.

### 3.3 Combination with concurrent chemoradiotherapy

For stage III unresectable NSCLC, consolidation immunotherapy followed by concurrent chemoradiotherapy (CCRT) is the gold standard of care (72). Radiation pneumonitis (RP) and pulmonary fibrosis are the two stages of radiation lung injury (RILI), a common side effect of radiation therapy (RT) in the chest (73, 74). One of the major barriers to the use of consolidation immunotherapy following CCRT is RP, which has a detrimental effect on survival and quality of life in patients with locally advanced NSCLC (LANSCLC). The GASTO-1043 study, a Phase II clinical trial, was explored the efficacy of Tα1 in the treatment of RP in patients with LANSCLC receiving CCRT (66). 138 patients with LANSCLC in total were enrolled in the study, 69 of whom received CCRT alone in the control group and 69 of whom received Tα1 treatment from the start of CCRT to 2 months after it ended in the experimental group. Compared to the control group, the experimental group had a lower incidence of grade 2 or higher RP (36.2% vs. 53.6%, P=0.040), lower incidence of G3 to G4 lymphopenia (19.1% vs. 62.1%, P<0.001), and significantly higher total lymphocyte count (TLC) (0.51 k/mL vs. 0.30 k/mL, P<0.001). Patients with maximum C-reactive protein (CRP) ≥ 100 mg/L (13.8% vs. 29.7%, P = 0.029) and patients with 2 to 3 weeks during CCRT CRP ≥ 10 mg/L (23.5% vs. 48.5%, P = 0.003) were significantly less common in the experimental group. Patients with LANSCLC who used Tα1 during and after CCRT showed lower rates of grade 2 RP and G3 to G4 lymphopenia compared to the control group, as well as higher TLC levels and lower CRP, demonstrating that Tα1 can treat side effects and immune dysfunction brought on by CCRT. Additionally, patients receiving Tα1 had a longer median progression free survival (PFS) (14.4 months vs. 10.7 months, P=0.131) and a longer median OS (34.8 months vs. 28.7 months in the control group, P=0.062). Despite being superior to the control group in both OS and PFS, however, the Tα1 experimental group did not achieve a statistically significant difference, which may be related to the smaller sample size.

### 3.4 Combination with targeted therapy

Patients with LUAD who have an EGFR target mutation that is positive can benefit from EGFR-tyrosine kinase inhibitors (TKI) targeted therapy, which is now one of the standard treatments for LC (75). In patients with IV-stage LUAD and EGFR mutations, some researchers have compared the clinical effects of EGFR-TKI monotherapy and EGFR-TKI plus thymosin therapy (67). To balance the baseline differences, propensity score matching (PSM) was carried out in a 2:1 ratio between the control group and the experimental group, with 130 LUAD patients being treated with first-generation EGFR-TKI (gefitinib/erlotinib/icotinib) and as part of the experimental group 65 LUAD patients being treated with EGFR-TKI plus thymosin (including Tα1 and thymopentin). The results revealed that the median PFS (14.4 months vs. 9.2 months, HR=0.433, P<0.0001) and median OS (29.5 months vs. 19.8 months, HR=0.430, P<0.0001) in the EGFR-TKI plus thymosin group were both significantly longer than those in the EGFR-TKI group, respectively. These findings demonstrated that EGFR-TKI plus thymosin was extremely effective in lowering the risk of relapse and improving prognosis in patients with LUAD compared with EGFR-TKI alone. The safety of EGFR-TKI plus thymosin was acceptable because there was no significant difference in ORR and DCR between the two groups, nor was there any significant difference in side effects. The number of CD3^+^T cells and the number of CD3^+^CD4^+^T and CD3^+^CD8^+^T subsets after treatment were significantly reduced in the EGFR-TKI monotherapy group (P<0.05), but there was no difference between these parameters in the EGFR-TKI plus thymosin group, indicating that the addition of thymosin based on targeted therapy has some effect on restoring the body’s immune activity. Further subgroup analysis revealed that in most subgroups, including age, EGFR mutation type (exon 19 deletion or exon 21 L858R mutation), presence or absence of bone metastases, central nervous system metastases, adrenal metastases, multiple lung metastases, pleural metastases, gefitinib or erlotinib, EGFR-TKI plus thymosin demonstrated a superior PFS advantage over EGFR-TKI monotherapy. There was no statistically significant difference in OS or PFS between the EGFR-TKI plus thymosin group and the single-agent group in patients with ECOG score of 2 who had liver metastases, received icotinib, and received EGFR-TKI as second-line therapy. Male patients could benefit from combination therapy in OS, and patients with LUAD who received radiotherapy solely for bone metastases benefited from combination therapy in PFS(P=0.041), while patients with LUAD who received radiotherapy for the remaining causes did not benefit in terms of OS or PFS. A comparison of the two thymosin medications revealed no statistically significant difference in PFS or OS between Tα1 and thymopentin, suggesting that the use of both thymosin medications in combination with an EGFR-TKI may be possible.

### 3.5 Postoperative adjuvant therapy

Because it boosts human immunity and enhances patients’ quality of life after LC surgery, Tα1 is frequently used in clinical practice as adjuvant therapy for postoperative LC patients. The long-term survival effects of Tα1 as postoperative immunomodulatory therapy were examined in a large, real-world study involving 5746 patients with NSCLC who underwent surgery and achieved R0 resection (68). The study compared 1027 operable (stage I–IIIA) NSCLC patients who received thymosin treatment to 1027 patients who did not receive thymosin treatment using PSM analysis. When compared to the control group without Tα1 treatment, the use of Tα1 in postoperative patients can lower the risk of recurrence and death in NSCLC patients. Even after multivariate analysis, Tα1 treatment still showed very significant differences in DFS (HR=0.655, P<0.0001) and OS (HR=0.548, P<0.0001). Additionally, following PSM, the Tα1 treatment group outperformed the control group in terms of 5-year DFS (77.3% vs. 64.7%, P<0.0001) and 5-year OS (83.3% vs. 72.7%, P<0.0001). Tα1 therapy may be an efficient postoperative adjuvant therapy in the early stage, especially in patients with stage IA NSCLC, as evidenced by the fact that the benefits of OS and DFS in patients with stage IA were more advantageous than those of all other stages in terms of staging (P<0.0001). According to pathological classification, other non-adenocarcinoma and non-squamous cell carcinoma subtypes were independently linked to worse OS (HR=2.019, P=0.0009) and DFS (HR=1.706, P=0.0038). The results of the subgroup analysis showed that patients who used Tα1 for more than 24 months had the best OS and DFS efficacy. The treatment effect was proportional to the length of treatment, with the best effect for more than 24 months, followed by treatment for 12–24 months, and the worst effects for less than 12 months. The 5-year DFS rates of the three groups were 84.7%, 81.0%, and 66.1%, respectively, and the 5-year OS rates were 92.2%, 83.7%, and 64.5%. This suggests that to reap the greatest benefits, patients with NSCLC may use Tα1 for longer than 24 months. Tα1 could show efficacy in OS and DFS in patients of any age, sex, Charlson Comorbidity Index (CCI), smoking status, and any stage of pathologic TNM, as well as patients with non-squamous cell carcinoma NSCLC who did not receive targeted therapy and BMI<28 kg/m^2^. This was confirmed by subgroup analysis. Tα1 only demonstrated OS efficacy in postoperative NSCLC patients who received chemoradiotherapy, and it did not affect OS or DFS in patients with a BMI>28 kg/m^2^, the pathological type of squamous cell carcinoma, targeted therapy, or combination therapy (including targeted therapy plus chemotherapy, targeted therapy plus chemoradiotherapy). Tα1 can be used as an adjuvant therapy after surgery to lower the risk of recurrence and increase the long-term survival of LC patients as well as an immunomodulatory therapy to enhance the postoperative quality of life of LC patients.

### 3.6 Combination with immunotherapy

Immunotherapy, an emerging therapeutic approach in recent years, has not only improved patient prognosis but also significantly altered the initial treatment regimen of LC patients. In the field of LC, immune checkpoint inhibitors (ICIs) that represent PDL1/PD-1 have the most applications and the best efficacy (76). The gold standard for immunotherapy in patients with advanced NSCLC is high PDL1 expression (PD-L1 Tumor Proportion Score 50%). Patients with NSCLC with high PDL1 expression can achieve better immunotherapy outcomes (77), this has also been confirmed by many RCTs (78, 79). Patients with high PDL1 expression in their NSCLC may respond better to immunotherapy. After long-term follow-up, a study of patients with metastatic melanoma who were given Tα1 discovered that those who also received the CTLA-4 ICI ipilimumab had an advantage in overall survival (80), this suggests that Tα1 and ICIs work in concert. It is expected that the combination of Tα1 and ICIs, represented by PD-1 inhibitors, may also have a beneficial synergistic effect on the treatment of LC. The prognosis of LC patients can be significantly improved by ICIs, but their clinical use is constrained by a few drawbacks. First off, a large number of LC patients are unable to respond to ICIs treatment effectively because some LC patients belong to cold immune tumors (such as immune-desert type and immune-excluded type), which lack lymphocyte infiltration (81, 82). If cold immune tumors can be transformed into hot immune tumors, this will greatly expand the clinical application range of ICIs and increase their clinical efficacy. The degree of immune cell infiltration in the tumor microenvironment (TME) is the primary distinction between cold immune tumors and hot immune tumors. NK cells and CD3^+^, CD4^+^, and CD8^+^T lymphocytes are currently thought to be the primary immune cells in the TME, with CD8^+^T lymphocytes playing a significant role in tumor killing (83, 84). Tα1, as an immunomodulator of thymic hormones, belongs to active immunity (18). As previously stated, using Tα-1 to treat LC can increase CD8^+^T cell counts, restore NK cell activity, and activate T lymphocyte immune function by increasing CD86 expression. When chemotherapy was combined with thymosin, more CD4^+^, CD8^+^, CD3^+^, and NK cells were present. Additionally, the CD4^+^/CD8^+^T cell ratios were higher than when chemotherapy was used alone. Thymosin also has the effect of increasing the helper T cell counts that have been decreased as a result of radiotherapy. Thymosin was also added to address the negative effects of EGFR-TKI treatment on the quantity of CD3^+^T cells produced by LC patients as well as the number of CD3^+^CD4^+^T and CD3^+^CD8^+^T subsets. In addition to improving Tα1 targeting, Tα1-RGDR promoted CD4^+^ and CD8^+^T cell infiltration of LC tissues and increased the immune activity of T cells by raising levels of IL-2 and IFN-γ. These findings imply that Tα1 can increase CD4^+^T, CD8^+^T, CD3^+^CD4^+^T, and NK cell numbers in LC patients. Tα1 has the potential to convert LC patients with cold immune tumors to hot immune tumors by increasing the cellular level and cell activity of immune cells like CD8^+^T cells, enhancing the clinical effectiveness of ICIs. Similarly, Tα1 can inhibit the migration and invasion of NSCLC with high expression of PD-L1 (31) and a favorable effect on immune cells, suggesting that the combination of Tα1 and ICIs may enhance the clinical effect of ICIs and even increase the proportion of LC patients who have an effective response to ICIs. Second, immunotherapy with ICIs is associated with adverse effects such as colitis and/or diarrhea (85). Studies have shown that Tα1 can enhance the small intestine tissue structure and barrier function of mice with cystic fibrosis (86). Additionally, a study has shown that Tα1 can successfully prevent ICIs-induced colitis in preclinical models by fostering the immune pathway of indoleamine 2,3-dioxygenase 1-dependent tolerance (87). These studies demonstrate that Tα1 has reduced the side effects of ICIs on the digestive system.

## 4 Discussion

Although the potential benefits of Tα1 in combination with immunotherapy in the treatment of LC patients have been outlined above, some issues still need to be resolved before actual clinical use. Numerous studies have shown that Tα1 increases the number and immune activity of immune cells in LC patients, but more basic studies are required to elucidate the detailed regulatory mechanism. The degree to which Tα1 promotes TME lymphocyte infiltration in LC is another area that needs to be studied more in the future, but there is currently a dearth of research in this area. However, this hypothesis needs to be confirmed in further clinical studies. The current clinical evidence is insufficient, and before they can be cautiously applied to clinical practice, exploratory clinical trials with the support of basic research conclusions must be conducted for LC patients treated with ICIs because the addition of Tα1 may also lead to immune dysfunction and interfere with immunotherapy. Tα1 has demonstrated the ability to reduce immunotherapy-related adverse reactions in addition to the potential to enhance the efficacy and effectiveness of the response of LC patients to immunotherapy. However, the current basic research is momentarily insufficient, necessitating more extensive basic research and preclinical research on the precise mechanism of Tα1 and ICIs in combination therapy for LC and immunotherapy-related adverse reactions, which is also one of the possible research directions of Tα1 in the future. In the field of immunotherapy, NK cells have demonstrated effective anti-tumor immunity in addition to ICIs, whether through their immune activity, adoptive cell therapy of NK cells, or even the use of CAR-NK cell therapy (88, 89). This article summarizes how Tα1 affects NK cell activity to function as an inhibitor of LC growth and metastasis. An in-depth investigation of the precise mechanism by which Tα1 enhances NK activity in LC models following chemotherapy, as well as the precise mechanism by which Tα1 and IFNs have a synergistic effect, is a very valuable research direction. Additionally, determining whether Tα1 and CAR-NK have a synergistic effect in the treatment of LC is another important research question.

The side effects of chemotherapy, radiotherapy, surgery, targeted therapy, and CCRT can all be significantly reduced with Tα1. The impact of Tα1 on reducing complications in LC patients who receive CCRT plus immunotherapy consolidation is something to look forward to because it makes it possible to perform consolidation immunotherapy after CCRT by reducing complications like RP associated with CCRT. The combination of Tα1 and chemotherapy for LC has the most studies, both basic and clinical studies, and the conclusion that Tα1 increases efficacy and reduces the toxicity of chemotherapy is firm. However, the chemotherapy regimens in these studies either include LC patients receiving NP/GP regimens or use cyclophosphamide to treat animal models, Tα1 and other chemotherapy drugs have not yet been supplemented. The activation of MDSCs’ immunosuppressive activity by Tα1 monotherapy is one of the mechanisms that Tα1 monotherapy can inhibit anti-tumor immunity. It is also proposed that Tα1’s anti-tumor efficacy can be restored by blocking the MyD88 signaling pathway, raising the possibility that future research may examine ways to mitigate Tα1’s adverse effects when used to treat LC. The specific mechanism of potential side effects when Tα1 is combined with targeted therapy drugs and immunotherapy drugs, as well as the investigation of whether Tα1 has other side effects, are also worthwhile research areas because prior basic research has only combined chemotherapy drugs. Although baseline differences were balanced using PSM analysis in the large real-world study that showed that Tα1 significantly improved OS and PFS in patients with LUAD undergoing surgery, this is retrospective, and prospective clinical trials are still required to provide higher-level evidence-based medical evidence. Tα1 as an immunomodulator can improve PFS and OS and reduce postoperative complications in patients with LC, but it is unclear what effect of Tα1 in the short term after LC surgery. A new perioperative management strategy called “enhanced recovery after surgery” (ERAS) seeks to improve surgical outcomes and quality of life by reducing postoperative complications and hospital stays in surgical patients while also speeding up their recovery and rehabilitation (90). ERAS was initially mainly used in colorectal surgery (91), the ERAS concept however, has become increasingly popular in recent years among patients undergoing thoracic surgery, especially pneumonectomy (92). The long-term OS and PFS benefits of Tα1 in LC patients have been confirmed, and Tα1 also has the potential to reduce complications, restore immune function, and improve quality of life in the short term after surgery, so future clinical studies are needed to determine whether Tα1 can be used postoperatively to further enhance the effect of ERAS.

We discovered that some studies had some restrictions and flaws. Meta-analyses had the highest level of evidence-based evidence, however, the quality of meta-analyses depended on the quality of the studies included in the analyses, which could vary widely from meta-analysis to meta-analysis (93). Although both meta-analyses decided to include only RCTs, many of the trials included in the studies were of poor methodological quality, except a small number of studies that explicitly proposed the use of the envelope method as a randomization regimen, and the remaining studies did not report methods of randomization. Two meta-analyses assessing the effects of thymosin in combination with chemotherapy were reviewed above. There were differences in some variables’ heterogeneity and the timing of index tests across studies. In addition, compared to typical phase III RCTs, the RCTs included in these two meta-analyses still have several flaws, most studies are small studies, most studies only involve not more than 100 patients, and there are few trials and samples that can be used to analyze survival. None of the trials reported PFS, these two meta-analyses of RCTs concluded that they did not reach the highest level of evidence because the majority of studies lacked NCT numbers, and the quality of the primary and secondary outcomes was moderate to very low according to GRADE grading. These shortcomings may be related to the earlier publication years of about half of the included studies. As the saying goes, “We can’t make bricks without straw,” these randomized studies have been able to demonstrate the efficacy of thymosin-combined chemotherapy in treating LC, and higher-quality meta-analyses need to be updated following the completion of well-designed large-scale RCTs.

Additionally, we discovered that Tα1 usage duration, dosage, and frequency varied significantly between studies. Continuous injection of Tα1 for more than 24 months had the best therapeutic effect in studies of Tα1 as a postoperative adjuvant therapy for LC patients, but there were significant differences in the duration of Tα1 use in other studies, such as clinical trials investigating the therapeutic effect of Tα1 on CCRT-induced side effects, which were used from the beginning of receiving CCRT to 2 months after the end of CCRT. These differences may be because different treatments often correspond to different clinical stages. Although the goal of adding Tα1 to various treatments varies, it is necessary to standardize the time of Tα1 usage for treatments. The majority of studies used Tα1 twice weekly, while a small subset used it on a weekly schedule. Tα1 usage frequency was also inconsistent. The dosage of Tα1 is typically 1.6 mg, and 1.6 mg per time is the current mainstream usage of Tα1. However, the use scheme is still somewhat unclear, necessitating additional research to determine the best treatment strategy and implement unified, standardized treatment.

Conclusions from various clinical studies disagree with one another (66, 68). In the GASTO-1043 study, LC patients receiving CCRT failed to achieve a statistically significant median OS d1ifference after adding Tα1 (34.8 vs. 28.7 months, P=0.062), and in a retrospective analysis examining the function of Tα1 as postoperative adjuvant therapy, LC patients receiving chemoradiotherapy could benefit from OS after Tα1 as adjuvant therapy. The difference between the conclusions of the two studies may be that patients with LC who received Tα1 as postoperative adjuvant therapy underwent surgery, and patients with improved treatment efficacy and could receive surgery had an earlier stage of TNM, and the GASTO-1043 study included patients with advanced LC, had a relatively small sample size of 138 patients, and had relatively insufficient postoperative follow-up time, which may have contributed to the failure to achieve a statistically significant difference in OS after the addition of Tα1 to CCRT. To determine the OS benefit of Tα1 in LC patients receiving CCRT, phase III clinical trials with longer follow-up times are required.

The combination of thymosin and targeted therapy is also controversial. According to the studies reviewed above, the combination of thymosin and EGFR-TKIs showed superior benefits for OS and DFS compared to EGFR-TKI monotherapy (67), whereas postoperative adjuvant therapy with Tα1 plus EGFR-TKIs (including targeted plus chemotherapy, targeted plus chemoradiotherapy) did not show better benefits in OS and DFS in LC patients with LC who were adjuvant with Tα1 plus EGFR-TKIs (68). As postoperative patients with LC treated with adjuvant thymosin were stage I-IIIA and primarily stage I, whereas the other study included LUAD patients in the analysis were stage IV, the different effects of EGFR-TKIs in combination with thymosin may be related to staging. Additionally, LC patients received first-generation EGFR-TKI in studies of thymosin combined with targeted therapy, and variations in staging and EGFR-TKIs may be the reason why the findings of the two studies are completely dissimilar. Two studies investigating the combined use of EGFR-TKIs and thymosin included 130 and 112 patients, respectively, and both are retrospective studies. The level of evidence-based medical evidence is not sufficient, and future prospective studies with larger sample sizes are needed to determine the actual benefits of EGFR-TKIs combining thymosin to treat LC in OS and DFS.

The side effects of thymosin drugs must also be taken into consideration in addition to their beneficial effects. Severe allergic reactions have been linked to thymopolypeptides injection. Thymopolypeptides injection is a mixture of peptides extracted from the thymus of animals, and complex peptide components are thought to be the main contributor to allergic reactions. Even though the thymosin drugs used in the studies discussed in this article are STPs with a single composition, it is still important to be aware of their side effects to prevent serious adverse events. It is not necessary for all LC patients to use thymosin drugs because the immune system is constantly in a state of dynamic balance and attempting to improve immunity will not necessarily result in a healthier body. The most fundamental requirement for safe drug use is a strict understanding of the indications, so using thymosin drugs blindly is not desired.

Thymosin drugs are more widely used for Tα1 and thymopentin, the conclusions of Tα1 were largely consistent across studies, but thymopentin was not. When combined with chemotherapy or EGFR-TKI, thymopentin has been shown to increase the number of T lymphocytes and NK cells in LC patients. Thymopentin in combination with chemotherapy did not demonstrate superior effects to chemotherapy alone in terms of ORR and DCR in the meta-analysis examining the effects of sTPs in combination with chemotherapy (64). However, there is a clinical advantage for OS when thymopentin and EGFR-TKI are combined (67). Thymopentin combined with various treatment modalities may have different therapeutic effects. These two studies are only retrospective, the level of evidence-based medical evidence is low, and the precise conclusions need to be proven by future high-level clinical trials. Additionally, there is a lack of basic and clinical trial research on the use of thymopentin in the treatment of LC. Therefore, before clinical promotion, there must be adequate evidence-based medical evidence and basic experimental support. In addition to Tα1 and thymopentin, other thymosins are also closely related to LC. Tβ4 is highly expressed in NSCLC tissues, as was previously mentioned, and part of the mechanism by which Tβ4 promotes LC progression has been discovered (94). Tβ4 high expression is associated with a poor prognosis, and its ability to promote cancer has also been demonstrated in gastric cancer and colorectal cancer (95, 96). Then, for Tβ4 as a target, one of the future research directions is the development of related drugs as well as the ongoing, in-depth study of the mechanism of Tβ4 promoting LC.

## 5 Conclusion

Thymosin is known to inhibit LC growth, proliferation, invasion, and migration as well as have immunomodulatory effects by controlling the quantity, activity, and function of NK cells and T lymphocytes, according to a large number of basic experiments. The findings of these studies lend some support to the clinical application of thymosin in LC patients. However, the findings of current basic studies on the addition of combination treatment are all based on preclinical chemotherapy models. This means that the current basic research does not include the evaluation of the combined effects of Tα1 and radiotherapy, CCRT, targeted therapy, and immunotherapy, and it is necessary to improve these preclinical models to provide theoretical support for the combined application of Tα1 and real-world clinical treatment of LC. Although the thymosin drugs represented by Tα1 have some results in the treatment of LC, they urgently require additional in-depth, creative basic research and higher-level evidence-based medical evidence support, both in basic experiments and clinical efficacy.

Finally, we anticipate further large-scale and carefully-planned prospective clinical trials to provide a higher level of evidence-based medical evidence support for the use of thymosin drugs in the field of LC, as well as an increasing number of in-depth basic studies to further reveal the specific mechanism of thymosin drugs for LC.

## Author contributions

YL wrote the original draft, completed the visualization, JL reviewed and revised the manuscript, YL and JL both conceived and designed the study. All authors read and approved the final manuscript. All authors contributed to the article and approved the submitted version.
